# *E. coli* nitroreductase NfsA is a reporter gene for non-invasive PET imaging in cancer gene therapy applications

**DOI:** 10.7150/thno.46826

**Published:** 2020-08-21

**Authors:** Alexandra Marie Mowday, Janine Naomi Copp, Sophie Philippa Syddall, Ludwig Jerome Dubois, Jingli Wang, Natasja Gabi Lieuwes, Rianne Biemans, Amir Ashoorzadeh, Maria Rosaria Abbattista, Elsie May Williams, Christopher Paul Guise, Philippe Lambin, David Francis Ackerley, Jeff Bruce Smaill, Jan Theys, Adam Vorn Patterson

**Affiliations:** 1 Auckland Cancer Society Research Centre, University of Auckland, Auckland, New Zealand.; 2 Maurice Wilkins Centre for Molecular Biodiscovery, School of Biological Sciences, University of Auckland, Auckland, New Zealand.; 3 School of Biological Sciences, Victoria University of Wellington, Wellington 6012, New Zealand.; 4 The M-Lab, Department of Precision Medicine, University of Maastricht, Maastricht, The Netherlands.; 5 Current address: The M-Lab, Department of Precision Medicine, University of Maastricht, Maastricht, The Netherlands.; 6 Current address: Michael Smith Laboratories, University of British Columbia, Vancouver, BC, V6T 1Z4, Canada.; 7 Current address: Walter and Eliza Hall Institute of Medical Research, 4 Research Avenue, Bundoora, Victoria, 3083, Australia.

**Keywords:** reporter gene imaging, PET imaging, nitroreductase, gene therapy, drug repurposing

## Abstract

The use of reporter genes to non-invasively image molecular processes inside cells has significant translational potential, particularly in the context of systemically administered gene therapy vectors and adoptively administered cells such as immune or stem cell based therapies. Bacterial nitroreductase enzymes possess ideal properties for reporter gene imaging applications, being of non-human origin and possessing the ability to metabolize a range of clinically relevant nitro(hetero)cyclic substrates.

**Methods:** A library of eleven *Escherichia coli* nitroreductase candidates were screened for the ability to efficiently metabolize 2-nitroimidazole based positron emission tomography (PET) probes originally developed as radiotracers for hypoxic cell imaging. Several complementary methods were utilized to detect formation of cell-entrapped metabolites, including various *in vitro* and *in vivo* models to establish the capacity of the 2-nitroimidazole PET agent EF5 to quantify expression of a nitroreductase candidate. Proof-of-principle PET imaging studies were successfully conducted using ^18^F-HX4.

**Results:** Recombinant enzyme kinetics, bacterial SOS reporter assays, anti-proliferative assays and flow cytometry approaches collectively identified the major oxygen-insensitive nitroreductase NfsA from *E. coli* (NfsA_Ec) as the most promising nitroreductase reporter gene. Cells expressing NfsA_Ec were demonstrably labelled with the imaging agent EF5 in a manner that was quantitatively superior to hypoxia, in monolayers (2D), multicellular layers (3D), and in human tumor xenograft models. EF5 retention correlated with NfsA_Ec positive cell density over a range of EF5 concentrations in 3D *in vitro* models and in xenografts *in vivo* and was predictive of *in vivo* anti-tumor activity of the cytotoxic prodrug PR-104. Following PET imaging with ^18^F-HX4, a significantly higher tumor-to-blood ratio was observed in two xenograft models for NfsA_Ec expressing tumors compared to the parental tumors thereof, providing verification of this reporter gene imaging approach.

**Conclusion:** This study establishes that the bacterial nitroreductase NfsA_Ec can be utilized as an imaging capable reporter gene, with the ability to metabolize and trap 2-nitroimidazole PET imaging agents for non-invasive imaging of gene expression.

## Introduction

Non-invasive reporter gene imaging is an indirect method to detect the process of gene expression inside cells 1. One of the major translational applications of reporter gene imaging is the ability to monitor quantitatively the spatial and temporal distribution of gene therapy vectors as a surrogate of imaging transgene expression 1-3. In addition, reporter genes can also be used to non-invasively and repetitively monitor adoptive cell-based therapies, providing the opportunity to study the cell trafficking, targeting, proliferation, and persistence of transplanted and stem/progenitor cells 2, 4. Early examples of reporter gene imaging involved optical methodology such as fluorescence 5 or bioluminescence 6, but more recently radionuclide-based methods have been developed following increased interest in positron emission tomography imaging (PET).

PET is a highly sensitive imaging method that takes advantage of coincident detection of two opposing 511 KeV gamma rays, emitted when positrons collide with electrons 7. This annihilation event allows precise positional detection and quantification of positron radiolabeled molecules, usually associated with a specific molecular probe or reporter gene 8. A general paradigm for non-invasive reporter gene imaging using radiolabeled probes was first described in 1995, whereby a reporter transgene (HSV1-tk) encodes an enzyme that selectively interacts with the probe (2-[^14^C]FIAU) to result in trapping and accumulation of radioactivity inside the transduced cell 9. Since then, several fluorinated ganciclovir analogues have been developed as novel probes to image HSV1-tk expression, including ^18^F-FHBG 10, 11. Preclinical safety evaluation of this probe has led to US FDA investigational new drug status 12 although potential toxicity associated with the systemic administration of nucleoside analogues and early degradation may limit signal integrity 13. The only clinically approved method available for reporter gene imaging is the human Sodium Iodide Symporter (hNIS), transfer of which allows visualization of the accumulated radioisotope ^124^I. Non-invasive imaging following viral gene transfer has been demonstrated as feasible in both preclinical animal models and humans 14-16, but it is possible that endogenous hNIS expression in the thyroid, stomach or other organs could limit sensitivity of the reporter assay in adjacent tissues 17.

Bacterial nitroreductase (NTR) enzymes are ideal candidates for reporter gene imaging applications, being of non-human origin and capable of metabolizing a diverse range of nitroheterocyclic substrates 18. NTR has been widely used in the context of gene-directed enzyme prodrug therapy (GDEPT), where sustained expression from either viral or bacterial vectors has produced significant therapeutic efficacy in combination with various prodrugs 19-21. In addition, NTR expression in zebrafish and rodent models in a tissue-restricted manner can provide chemically inducible single cell ablation, allowing studies of cellular function and regeneration 22-24. At present, NTRs are commonly imaged using fluorescent and near-infrared probes 25, 26 but tissue penetration of even the most effective near-infrared probes is typically attenuated beyond 1 cm, making this method unsuitable for routine clinical use 27. However, there are several well-studied nitroheterocyclic PET probes available at various stages of clinical development and validation for the imaging of hypoxia including the ^18^F-labeled 2-nitroimidazoles (2-NIs) fluoromisonidazole (F-Miso) 28, EF5 29, EF3 30 and HX4 31. These probes can penetrate into tumors to detect a subset of hypoxic cells and, following an oxygen-sensitive one-electron reduction step, reactive products are formed that bind covalently to cell components and cause intracellular accumulation of the 2-NI 32. We considered it possible that certain oxygen-insensitive (Type I) bacterial NTR enzymes might offer the potential to bypass the oxygen sensitive step to generate the reactive product directly by a concerted two-electron reduction mechanism 18. This is an attractive option given that all clinical-enabling processes are *a posteriori* achieved, negating the need for *de novo* development of a radionuclide PET probe to image NTR expression. The principle caveats are whether an NTR can be identified that is capable of metabolizing 2-NI substrates, and whether an NTR-expressing cell can be accurately quantified against a background of non-specific probe retention.

The NTR NfsB from *Escherichia coli* has been extensively studied as a model nitroreductase for gene therapy vectors (for a more comprehensive review, see Williams *et al*., 2015 18). However, the only study to date to report activity of NfsB with a 2-NI substrate describes this enzyme as being inactive with misonidazole (a non-fluorinated analogue of the hypoxia probe F-Miso) 33. Here we confirm that NfsB is ineffective at reducing 2-NI substrates. In contrast, we demonstrate that an alternative oxygen-insensitive nitroreductase from *E. coli*, NfsA (NfsA_Ec; UniProt P17117), exhibits a significant metabolic capacity for 2-NI agents, several of which have demonstrated clinical utility as hypoxia PET imaging agents. Cells expressing *nfsA*_Ec were shown to be efficiently labelled with the 2-NI EF5 in a manner that was dose-dependent and quantitatively superior to that of hypoxic activation, in monolayers (*in vitro* 2D), multi-cellular layers (*in vitro* 3D), and in human tumor xenografts (*in vivo* 3D). The presence of *nfsA*_Ec in tumor xenografts was also predictive of prodrug efficacy *in vivo*. Proof-of-principle microPET imaging studies were successfully conducted using ^18^F-HX4.

## Results

### NfsA_Ec is efficient at metabolizing 2-nitroimidazole compounds *in vitro*

**Figure 1:**
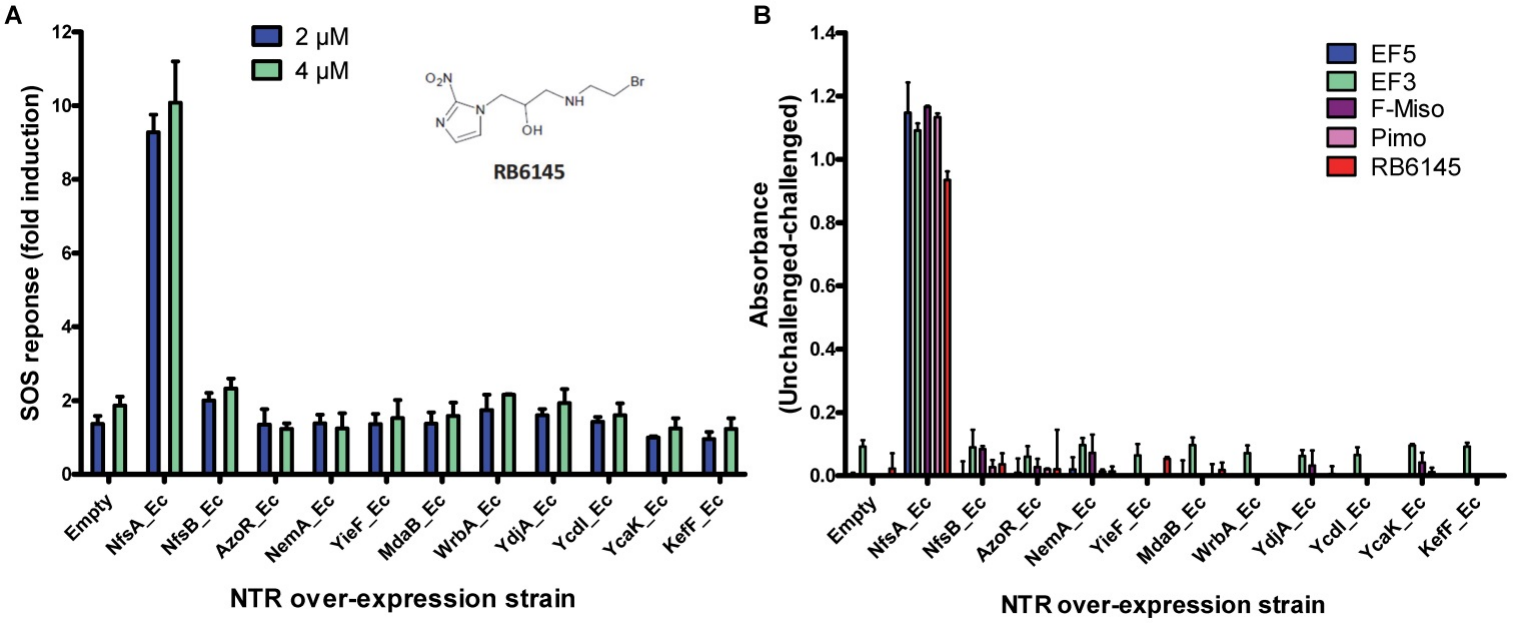
** A) RB6145 induced SOS response of the *E. coli* NTR over-expression library.** SOS-R2 NTR over-expression strains were grown as individual cultures in 96 well microplates and challenged with either 2 μM or 4 μM RB6145 for 4 hours prior to quantification of the SOS response induction by β-galactosidase assay. Fold induction is derived from the Miller units recorded on drug exposure divided by those of unchallenged replicate cultures. Data are the mean of two independent experiments ± SD. **B) NADPH consumption by SOS-R2::*ntr* cell lysates in the presence of 2-nitroimidazole compounds.** Crude cell lysates were incubated with 200 µM NADPH and 150 µM of each 2-NI test compound for 30 mins. Addition of NBT/PMS post-incubation yielded formazan dye in proportion to the residual NADPH, which was quantified by measuring absorbance at OD_590_. Plotted values indicate the extent of 2-NI metabolism by each NTR over-expressing strain and were derived by subtracting the OD_590_ of compound challenged lysates from the OD_590_ of unchallenged duplicate controls. Data are the average of two independent assays ± SD.

To determine whether NTR enzymes can successfully metabolize 2-NI compounds with clinical utility as PET imaging substrates, we over-expressed each of a previously identified panel of eleven candidate NTRs from *E. coli* 34 in a reporter strain of *E. coli*, SOS-R2, which expresses quantifiable β-galactosidase from an SOS (DNA damage response) promoter 35. We then challenged each over-expression strain with RB6145, a 2-NI prodrug that is converted to an alkylating agent following nitroreduction 36. The NfsA_Ec over-expressing strain, which exhibited a ~10-fold induction in SOS response following challenge with either 2 µM or 4 µM RB6145, was the only strain with a significantly different response from the empty plasmid control (*P* < 0.01, Figure 1A). To conduct a more comprehensive analysis of activity with diverse 2-NI substrates we subjected each NTR over-expressing *E. coli* strain to an NADPH depletion assay. We observed that crude lysates derived from the NfsA_Ec over-expressing strain consumed NADPH at a greatly heightened rate when incubated with any of the 2-NI substrates HX4, EF5, EF3, F-Miso, pimonidazole or RB6145 (Figure 1B). In contrast, NADPH consumption by crude lysates from the other NTR over-expression strains or the empty plasmid control was not increased in the presence of these substrates. When grown in liquid cultures, the NfsA_Ec over-expression strain was substantially more sensitive than the other strains to each of the 2-NI compounds, as quantified in IC_50_ assays (Table 1). The NfsB over-expression strain exhibited low-level sensitivity to HX4, F-Miso and EF3, but sensitivity of this strain to the remaining 2-NI compounds was above the detection threshold. None of the other strains had any detectable sensitivity to any of the 2-NI compounds tested. Consistent with these observations, the catalytic efficiency (*k_cat_/K_M_*) of purified NfsA_Ec protein was at least 40-fold greater than that of purified NfsB for every substrate tested, with the exception of HX4, with which NfsA_Ec was approximately 3-fold more efficient than NfsB (Table 2).

We next sought to investigate the performance of the different NTR candidates with 2-NI compounds within a human tumor cell environment. A previously generated panel of HCT116 cell lines individually over-expressing each NTR candidate 34 was screened using a surrogate endpoint of relative sensitivity to the panel of 2-NI compounds by anti-proliferative assay (Table 3). Again, significantly increased sensitivity to 2-NI substrates was observed exclusively in cell lines expressing NfsA_Ec or NfsB, in comparison to non-transfected wild type (WT) controls. Cells expressing NfsA_Ec were generally the most sensitive, with WT:NTR IC_50_ ratios (WT IC_50_/NTR IC_50_) ranging from 52-1600 fold (see Table S1 for actual IC_50_ values). In contrast, sensitivity of NfsB expressing cells to most 2-NI substrates was more modest (WT:NTR IC_50_ ratios between 2-203 fold). PR-104A was included as a nitroaromatic prodrug reference, with NfsA_Ec and NfsB (and to a lesser extent MdaB and NemA) demonstrating anti-proliferative activity consistent with our previous assessment of these enzyme activities in *E. coli* cell lines and as purified proteins 35.

### NfsA_Ec-dependent activation of EF5 is superior to hypoxia-dependent activation at low cell density

**Figure 2:**
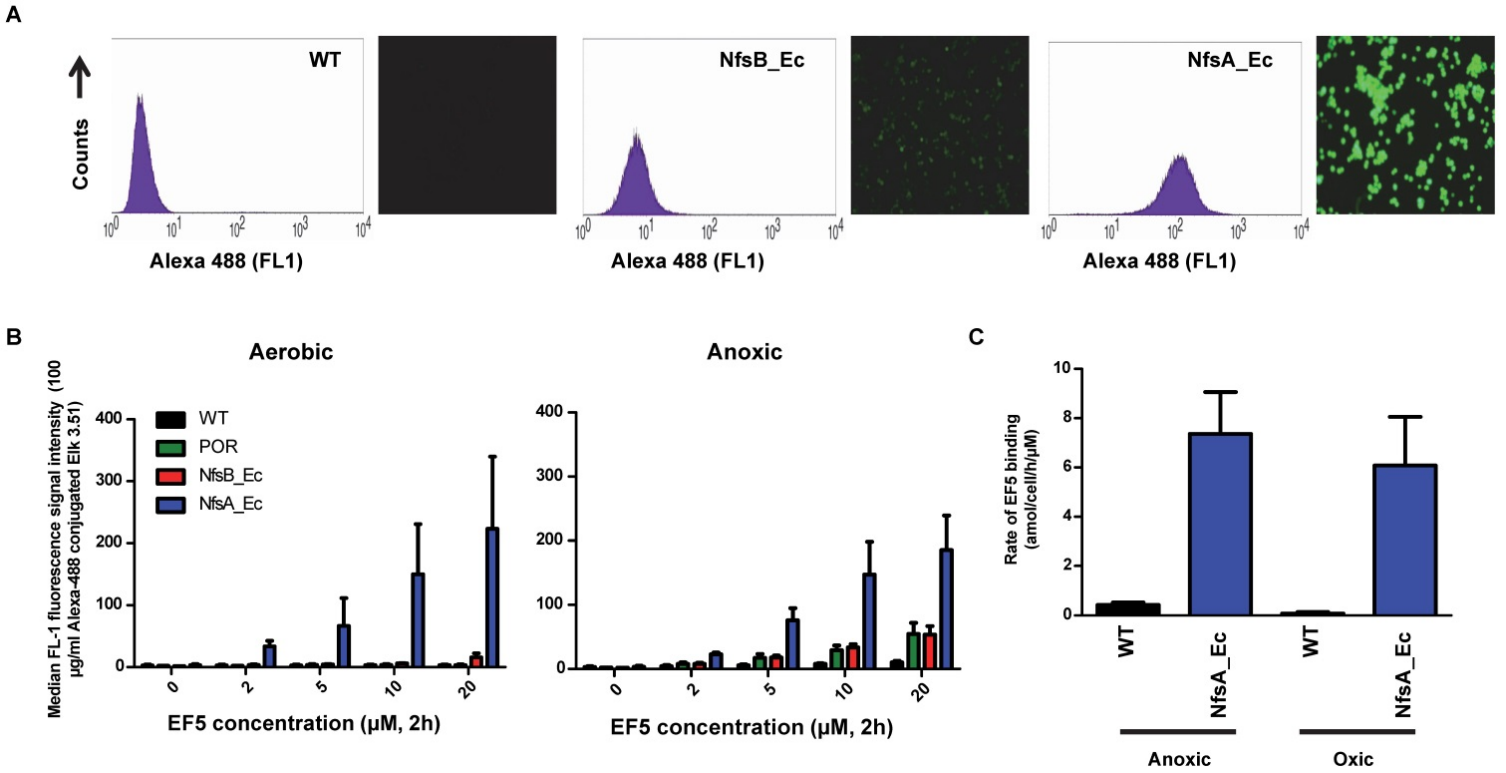
** NfsA_Ec dependent activation of EF5 is superior to hypoxia dependent activation at low cell density. A)** Flow cytometry analysis and fluorescent microscopy of nitroreductase-expressing HCT116 cells after 2 h aerobic exposure to 20 µM EF5. EF5 adducts were detected using a specific antibody conjugated to Alexa 488. **B)** Metabolism of EF5 after 2 h exposure in aerobic and anoxic conditions in parental HCT-116 cells and cells over-expressing NfsA_Ec, NfsB_Ec or POR. EF5 adducts were detected by flow cytometry using the Alexa 488 conjugated antibody. Values are mean ±SEM of two independent experiments. **C)** Rate of ^14^C-EF5 binding in HCT116 WT and HCT116 NfsA_Ec cells during a 2 mM exposure for 4 hours under aerobic or anoxic conditions.

EF5 was selected as the preferred 2-NI substrate for proof-of-principle studies into NTR activation due to the availability of well-validated methodology for quantitative adduct detection using a fluorescently-tagged monoclonal antibody 37. To establish if NTR activation of EF5 is superior to that of hypoxia-dependent activation (i.e. activation by one-electron reductases such as POR), NfsA_Ec and NfsB cells were exposed to EF5 and the ability to produce detectable adducts was first investigated (Figure 2). NfsA_Ec expressing cells provided the greatest increase in fluorescence intensity (and therefore EF5 adduct formation) in comparison to WT cells under aerobic conditions (Figure 2A), an observation consistent with the IC_50_ screen. NfsB expressing cells did provide some increase in fluorescence intensity, but to a much lesser degree than NfsA_Ec. Adduct formation under anoxic conditions was then quantified in these cell lines over a range of EF5 concentrations and compared to that of POR, a major one-electron reductase involved in the metabolism of hypoxia-activated prodrugs 38, 39 (Figure 2B). Our results clearly demonstrate that metabolism of EF5 and retention of adducts is concentration-dependent with respect to both one and two-electron reduction. Although POR and NfsB show an increase in adduct retention at 20 µM EF5 under anoxia in comparison to WT cells (5.7-fold and 5.3-fold respectively at 20 µM EF5), NfsA_Ec is far superior to both, demonstrating a 19-fold increase in fluorescence in the same conditions. To further demonstrate this, we examined the cumulative binding of ^14^C-EF5 over a 4-hour period under aerobic and anoxic conditions in WT and NfsA_Ec cells (Figure 2C). Under anoxia, this methodology confirmed that retention of ^14^C-EF5 adducts in NfsA_Ec expressing cells is significantly greater than observed in HCT116 WT cells (17-fold increase in ^14^C-EF5 binding, *P* < 0.01). Rates of ^14^C-EF5 binding in NfsA_Ec expressing cells were similar under both aerobic and anoxic conditions (*P* > 0.05).

### NfsA_Ec-dependent metabolism of EF5 is detectable at high cell density

**Figure 3:**
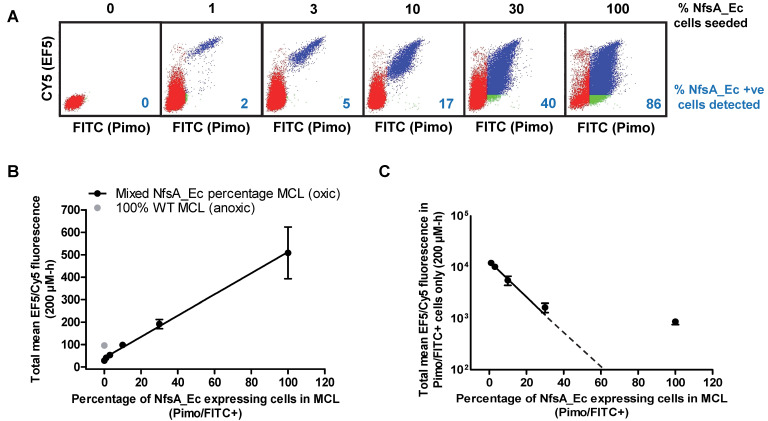
** Evaluation of NfsA_Ec dependent EF5 metabolism at high cell density. A)** Representative dot plots from each mixed NfsA_Ec MCL treated with 200 µmol-hr EF5. **B)** Total EF5/CY5 fluorescence in MCLs with varying densities of *nfsA*-expressing cells under oxic conditions compared to 100% HCT116 WT MCLs in anoxic conditions, *R^2^* = 0.99. Values are mean ±SEM of 3 independent experiments. **C)** Level of EF5/CY5 expression in pimonidazole positive (NfsA_Ec) cells only, *R^2^* = 0.80 (all data points) and* R^2^* = 0.99 (all but data point for 100% NfsA_Ec MCL). Values are mean ±SEM of 3 independent experiments.

In three dimensions (3D) it is possible that efficient metabolic consumption of EF5 may lower the available concentration at distal locations in a dense, multi-cellular environment 40, 41. Therefore, the influence of increased reductive metabolism of EF5 by NfsA_Ec was analyzed using high cell density multicellular layers (MCLs) (Figure 3). MCL populations containing a mixture of WT and NfsA_Ec-expressing cells were used, necessitating the development of a method to differentiate between the two cell types following completion of the experiment and dissociation of the MCL. Subsequent incubation with pimonidazole under aerobic conditions directly phenotyped the cell populations by exploiting the oxygen-insensitive catalytic activity of the NfsA_Ec-expressing cells (pimonidazole positive), distinguishing them from the WT cells (pimonidazole negative) also in the MCL (Figure S2).

MCLs were treated with EF5 under hyperoxic conditions (95% O_2_, 5% CO_2_) to prevent hypoxic activation by one-electron reductases. For MCLs with a small percentage of NfsA_Ec cells (1, 3 or 10% of cells seeded), two defined populations were observed; a pimonidazole negative (WT) population with reduced levels of EF5 binding was present (Figure 3A, red dots) in addition to a pimonidazole positive (NfsA_Ec) population with high levels of EF5 binding (Figure 3A, blue dots). A minor proportion of the pimonidazole negative cells was EF5 positive, indicating that diffusion of EF5 metabolites may occur from NfsA_Ec cells into surrounding WT cells.

At higher NfsA_Ec densities (30% and 100% of cells seeded), there was evidence of a pimonidazole positive cell population that was EF5 negative, consistent with extensive EF5 consumption leading to a lack of penetration into the MCL. (Figure 3A, green dots). However, of note, pimonidazole negative cells were also detected in MCLs that consisted of 100% NfsA_Ec expressing cells, possibly indicating a loss of metabolic capacity. Overall, the total mean EF5 fluorescence of all cells in the MCL correlated well with the NfsA_Ec positive cell subpopulation (*r^2^* = 0.99; *P* < 0.001, Figure 3B), demonstrating that EF5 detection is proportional to the NfsA_Ec positive cells present. However, the EF5 fluorescence of the NfsA_Ec positive cells alone deviated from linearity above approximately 30% NfsA_Ec cells suggesting the presence of a metabolic barrier (Figure 3C).

### EF5 metabolism can identify NfsA_Ec expressing cells in human tumor xenografts

**Figure 4:**
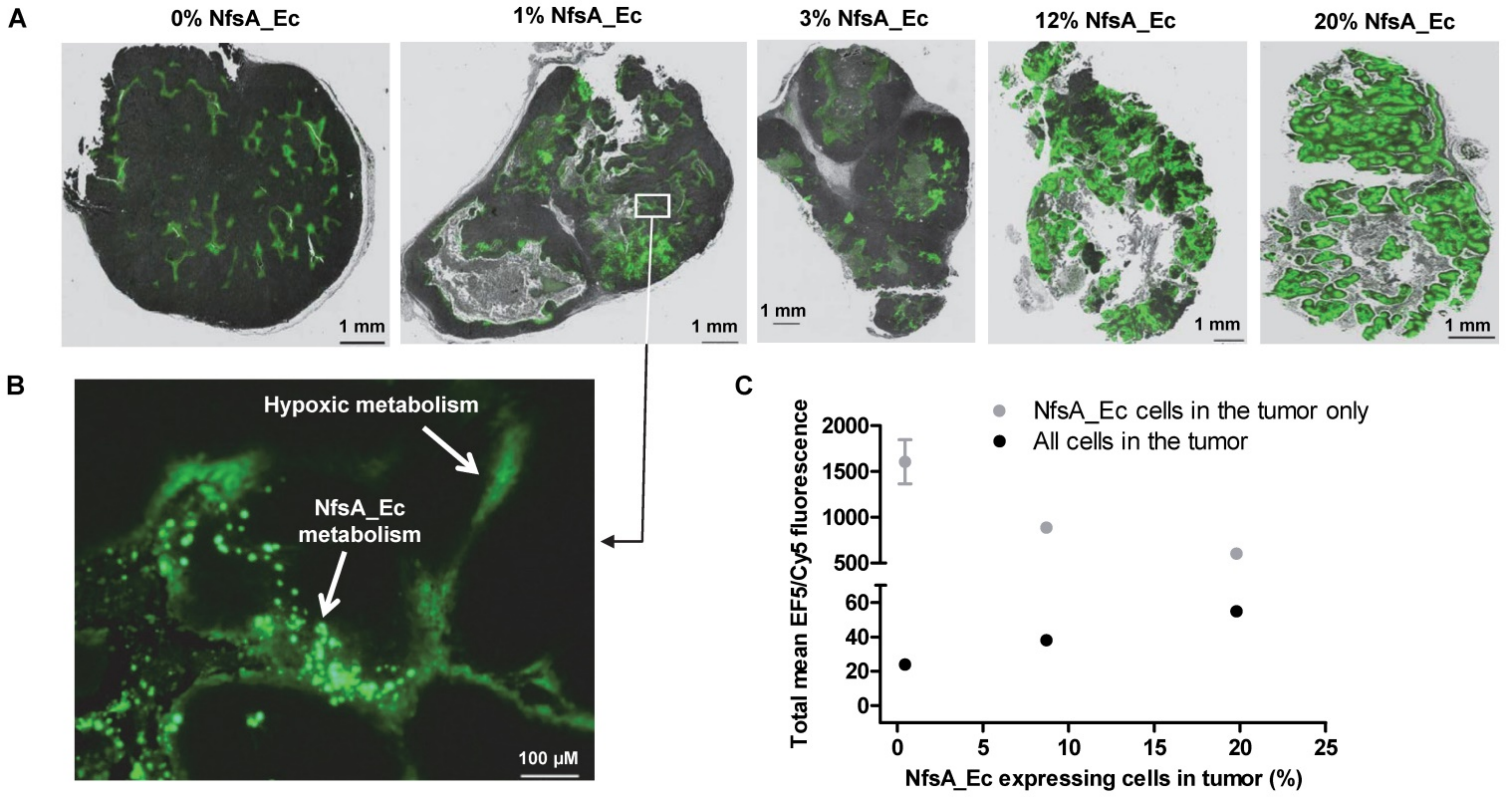
** EF5 labelling can be used to detect NfsA_Ec-expressing cells *in vivo*. A)** Intensity of EF5 staining in tumours consisting of a majority of HCT116 WT and a minority (1, 3, 12 or 20%) of HCT116 NfsA_Ec cells. EF5 adducts were detected using an Alexa 488 conjugated antibody, fluorescent images were overlaid onto adjacent sections stained with H&E. **B)** Magnification of a section of the 1% HCT116 NfsA_Ec xenograft. **C)** Intensity of EF5 labelling in mixed HCT116 WT/HCT116 NfsA_Ec xenografts. NIH-III mice bearing 300 mm^3^ tumours were treated with EF5 and 24 hours later tumours were excised, enzymatically dissaggregated, and 1 x10^6^ cells were treated with 20 µM pimonidazole for 1hr to label NfsA_Ec expressing cells *ex vivo*. EF5 adducts were detected using a specific antibody conjugated to Alexa 488. EF5 staining intensity relative to the percentage of NfsA_Ec expressing cells is shown for all cells in the tumour (*R^2^* = 0.99) and the pimonidazole positive NfsA_Ec-expressing population (*R^2^* = 0.90). Values are Mean ±SEM for 2-3 tumours/group.

Although high cell density MCLs are more informative than two-dimensional monolayers, these models lack key pharmacokinetic/pharmacodynamics features that are inherent to an *in vivo* setting. Therefore, the ability of EF5 to label NfsA_Ec expressing cells and the spatial distribution of the resulting EF5 adducts was assessed using tumor xenografts containing variable proportions of NfsA_Ec expressing cells (Figure 4). The spatial distribution of EF5 adducts throughout tumor sections was relatively homogeneous and, consistent with the MCL data, there did not appear to be EF5 consumption or penetration problems in xenografts with an NfsA_Ec proportion ≤ 20%, with large sections of the tumor demonstrating significant EF5 retention (Figure 4A). Lower intensity staining of the hypoxic regions was clearly evident, particularly in the peri-necrotic regions of the xenografts containing a low proportion of NfsA_Ec expressing cells, but it is clear that EF5 adduct retention in NfsA_Ec expressing cells was superior to EF5 adduct retention in hypoxic parental cells (Figure 4B; inset of the 1% NfsA_Ec xenograft). Upon dissociation of these tumors into single cell suspensions to determine the EF5 fluorescence of individual cells, a similar trend to the *in vitro* data (Figure 3) was observed; the percentage of Nfs_Ec expressing cells showed a positive correlation with the mean EF5 fluorescence for all tumor cells (*R^2^* = 0.99) and a negative correlation with the NfsA_Ec expressing cells (*R^2^* = 0.90). Overall, this indicates that EF5 dependent labelling of tumor cells is efficient when the frequency of NfsA_Ec-positive subpopulation is ≤ 20%.

### The presence of NfsA_Ec predicts for PR-104 efficacy *in vivo*

**Figure 5:**
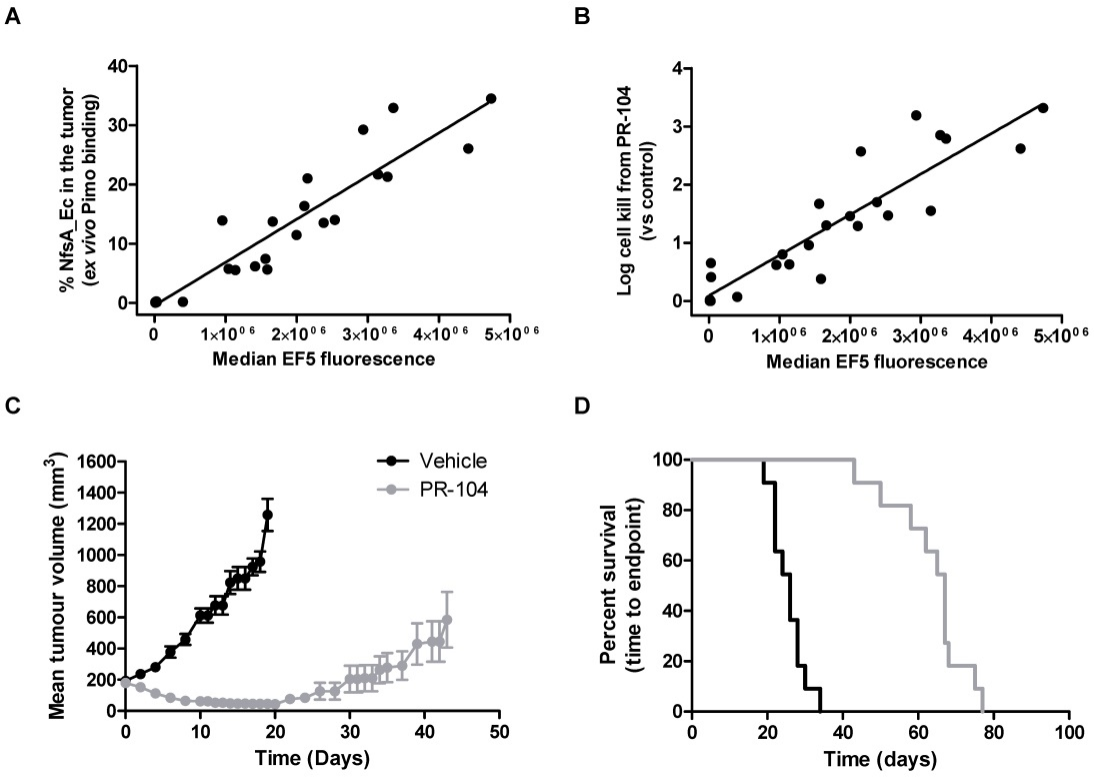
** The presence of NfsA_Ec is predictive of PR-104 efficacy *in vivo*. A and B)** The *in vivo* relationship between EF5 reduction, pimonidazole reduction and cell kill from PR-104. Tumours composed of 0-40% NfsA_Ec cells were grown in NIH-III mice and excised when the tumours reached a mean diameter of 10mm. Prior to excision, mice were dosed with 562 μmol/kg PR-104 followed by 30 mg/kg EF5 two hours later. Tumour cells were treated with 20 µM pimonidazole *ex vivo* for two hours before being labelled for both EF5 and pimonidazole adducts and plated to obtain a clonogenic endpoint for cell kill from PR-104. **C and D)** *In vivo* efficacy of PR-104 in 22% NfsA_Ec-expressing tumour xenografts. Average tumour volume and Kaplan-Meier survival plots for 100% WT and 22% NfsA_Ec/78% WT HCT116 xenografts grown subcutaneously on NIH-III mice and treated with PR-104 (1000 µmol/kg) or vehicle. N= 8-11 per group.

Next, we critically determined whether the proportion of NfsA_Ec positive cells in individual tumors correlated with the total EF5 signal, and whether this in turn could predict the therapeutic activity of the dual hypoxia/NfsA_Ec-activated prodrug PR-104 *in vivo*. Here animals bearing HCT116 tumors which were composed of a range of NfsA_Ec positive cells were treated with PR-104, followed by EF5 three hours later. Single cell suspensions of these tumors were isolated and employed to quantify median EF5 signal generated from both hypoxic and NfsA_Ec metabolism, and actual proportions of NfsA_Ec positive cells in the tumor at the time of treatment (*ex vivo* aerobic pimonidazole labelling). Median EF5 fluorescence of the entire tumor correlated with the percentage of NfsA_Ec positive cells in the tumor at the time of treatment (Figure 5A); increasing the proportion of NfsA_Ec positive cells (0% - 33%) yielded a detectable increase in median EF5 signal (*R^2^* = 0.87). This indicates *in vivo* EF5 signal arising from hypoxia only accounts for a small fraction of the variability and is consistent with the findings in figure 2B and 2C. Concurrently, PR-104 dependent tumor clonogenic cell kill was monitored by colony forming assay. Here, tumor cell kill following PR-104 treatment was greatly increased, exceeding 99% loss of viability and correlating with the quantum of EF5 signal (*R^2^* = 0.83, Figure 5B). Following this, we determined whether PR-104-dependent loss of tumor clonogenicity translated into significant tumor growth delay (TGD). We have previously shown that HCT116 wild type xenografts are refractory to single agent PR-104 in a growth delay setting 42. In this study, xenografts of the same genetic background that express a minority of NfsA_Ec cells (21.9% ± 2%) produced significant tumor regressions when treated with single dose PR-104 (Figure 5C). A TGD of 158% was generated, with the median survival endpoint of 67 days representing an increase of 41 days over untreated controls (Log rank, *P* < 0.001, Figure 5D).

### ^18^F-HX4 microPET establishes *in vivo* proof-of-principle

**Figure 6:**
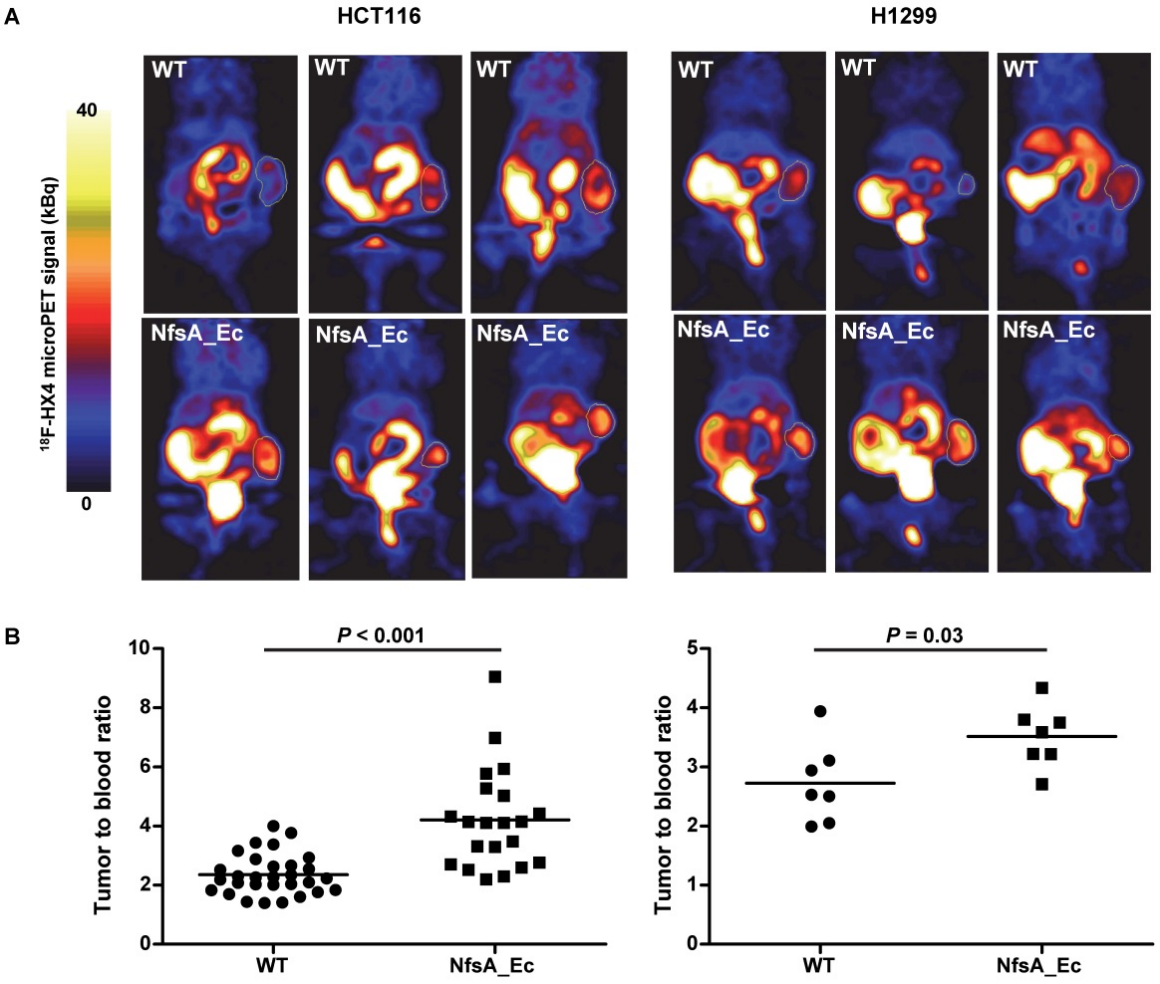
**^18^F-HX4 microPET establishes *in vivo* proof-of-principle. A)** Reconstruction of representative PET images two hours after tracer injection in HCT116 and H1299 xenografts. Tumours are delineated using a white line. Uptake of ^18^F-HX4 is higher in NfsA_Ec expressing tumours in comparison to WT. **B)** Quantification of tumour:blood ratios of the different experimental groups.

To establish whether PET imaging could be used as a tool to detect NfsA_Ec expression, two xenograft models (HCT116 and H1299), with or without NfsA_Ec overexpression, were treated with the 2-NI nucleoside analogue ^18^F-HX4 43 prior to imaging with microPET. ^18^F-EF5 could not be used in this setting due to requirement of ^18^F_2_ gas for radiolabeling by electrophilic fluorination. ^18^F-HX4 has been in clinical development for imaging of tumor hypoxia 31, 44 and is significantly more water soluble than ^18^F-EF5, clearing faster from non-hypoxic tissue 27, 45. In addition, NfsA_Ec had demonstrable anti-proliferative activity with HX4 (Table 3). A heterogeneous high accumulation of ^18^F-HX4 was observed in both tumor models (Figure 6 and Figure S3), with low ^18^F-HX4 levels in the surrounding normal tissues, except for the bladder and kidneys (the major route of excretion). This was not unexpected, given that ^18^F-HX4 is used as a hypoxia-marker, whereby the heterogeneous HX4 pattern in tumors is representing the hypoxic heterogeneity within the tumor microenvironment (hypoxic fraction HCT116 = 10-12%, H1299 = 1-7%). A significantly higher tumor-to-blood ratio was observed for the NfsA_Ec overexpressing tumors in both the HCT116 tumor model (4.21 ± 1.71 and 2.34 ± 0.68 for NfsA_Ec and parental tumors respectively; *P* < 0.001) and the H1299 tumor model (3.52 ± 0.52 and 2.73 ± 0.68 for NfsA_Ec and parental tumors respectively; *P* = 0.03), providing initial proof-of-principle for the ability to detect NfsA_Ec expression by non-invasive PET imaging.

## Discussion

Non-invasive reporter gene imaging has the potential to expedite clinical development in a variety of therapeutic settings, given recent progress in the use of biomarkers and companion diagnostics for drug development and treatment monitoring 46-48. For example, extensive invasive biopsy and blood sampling was used to monitor virus replication and transgene expression in Phase II clinical trials of Reolysin and Pexa-vec 49, 50. However, it is recognized that these methods are labor intensive and can be logistically and ethically challenging. In contexts such as these, the inclusion of a reporter gene that can metabolize and trap a PET imaging agent for non-invasive imaging of gene expression as a surrogate of vector deposition and spread may offer significant advantages. By screening and biochemically characterizing a collection of eleven *E. coli* nitroreductase candidates for activity with 2-NI substrates that have demonstrated clinical utility as hypoxia PET probes, NfsA_Ec was identified in this study as a possible candidate for radionuclide-based reporter gene imaging.

EF5 was initially selected for further evaluation in combination with NfsA_Ec as availability of a fluorescent antibody allowed for quantitative assessment of adduct detection. As standard hypoxic activation of EF5 will inevitably occur simultaneously with NfsA_Ec activation, it was important to demonstrate that that retention of EF5 adducts in NfsA_Ec positive cells was superior to retention in WT anoxic cells (Figure 2). Even in cases where cells were engineered for high-level overexpression of POR, a known EF5 reductase 51, these cells were still 4-fold less fluorescent than NfsA_Ec expressing cells. Notably there was no increase in fluorescence in anoxic NfsA_Ec cells compared to oxic NfsA_Ec cells, consistent with a high relative contribution of oxygen-insensitive NfsA_Ec metabolism versus that of human one-electron reduction.

A linear relationship is required if PET probe retention (and therefore NfsA_Ec activity) is to be used to quantify reporter gene expression non-invasively. It has previously been demonstrated that inefficient penetration of drugs into tissue-like structures can occur due to rapid metabolic consumption 52. Since NfsA_Ec was a demonstrably efficient metabolizer of EF5 (Figure 2B, Supplementary Table 1), the ability of EF5 to penetrate and label high cell density, mixed cell population MCLs was tested (Figure 3A). At this high cell density, a shift towards greater aerobic EF5 labelling in pimonidazole negative (i.e. WT) cells was observed as the percentage of pimonidazole positive (NfsA_Ec expressing) cells in the mixture increased, suggestive of a metabolic activation of EF5 in NfsA_Ec cells and subsequent metabolite distribution into surrounding WT cells. There was also evidence that increased reductive metabolism of EF5 by NfsA_Ec compromised penetration under conditions of excess NfsA_Ec positive cells (> 30%), as seen by the presence of a subset of pimonidazole positive (NfsA_Ec) cells that were not labelled by EF5 within the MCL. MCLs consisting of 100% NfsA_Ec cells showed evidence of a pimonidazole negative population, a phenotype normally associated with cells lacking NfsA_Ec expression. Several factors likely contribute to the decreased rates of EF5 labelling in NfsA_Ec cells with increasing concentrations of NfsA_Ec positive cells in the mixture. One explanation for this finding could be the loss of transgene expression in a proportion of cells. It is also possible that at 200 µmol-hr EF5, a concentration exceeding the EF5 IC_50_ of 5 µM (*ca.* 90 µmol-hr with 18 hours of exposure), cells which lie at the periphery of the MCL may no longer be viable at the time of pimonidazole labelling, or may show a marked reduction in metabolic capability owing to EF5 adduct toxicity (Figure 3C). Despite the caveats of the method described above, a positive linear correlation between the percentage of NfsA_Ec cells present in the MCL and the mean EF5 fluorescence for all cell populations was observed (Figure 3B).

Tumor xenograft data suggested that the large metabolic advantage of NfsA_Ec metabolism that was observed *in vitro* was also apparent *in vivo*. Using xenografts with an NfsA_Ec proportion ≤ 20% there was no apparent evidence of excessive EF5 consumption or marked penetration problems (Figure 4). Consistent with this interpretation, xenografts with up to 30% NfsA_Ec-positive cell populations displayed linearity with respect to median EF5 signal intensity (Figure 5A). When applied in a GDEPT setting, a 21% level of NfsA_Ec expressing cells in a tumor xenograft achieved significant clinical efficacy with PR-104, and the quantum of EF5 signal *in vivo* (and therefore proportion of NfsA_Ec positive cells) was also shown to predict the amount of cell kill achieved with PR-104 (Figure 5B).

A therapeutic enzyme such as NfsA_Ec that can coordinately metabolize PET imaging agents and bioreductive prodrugs in a GDEPT context may potentially be applied to a wide variety of different vector platforms. The cofactor (NAD(P)H) dependence of NfsA_Ec will limit activity to intact cells, possibly restricting the ability to image transgene expression in potent viral systems where accelerated lysis occurs. Imaging would only be biologically functional for a brief period during the active life cycle of the virus, and may not be entirely predictive of total intra-tumor viral titer. For example, differentiation between an inactive virus yielding minimal NTR activity and an active virus exhibiting rapid propagation and cell lysis may not be possible. Alternatively, monitoring of the active viral life cycle with exogenous substrates may instead confer an accurate measure of real-time viral behavior, as opposed to a method that determines cumulative lytic burden. This would not be a problem for bacterial vectors such as *Clostridium* species, which germinate and metabolize autonomously and do not cause cell lysis 53, making *nfsA*_Ec a particularly attractive transgene for gene therapy approaches using this vector platform. Elsewhere, we have reported that an NfsA_Ec variant engineered for improved metabolism of PR-104A appeared more effective in a bacterial rather than human cell environment 54, which would also support the use of bacterial vectors. Species of bacteria have previously been detected in tumors using microPET, where there was a linear correlation between the number of viable bacteria in the tumor and accumulation of the PET tracer 55, 56. This indicates that using a PET imaging approach to non-invasively image colonized tumors is possible, however further *in vivo* testing using the appropriate vector platform will be required for future development of this technology in a GDEPT context.

Immune responses against transduced cells could represent a major obstacle to the success of reporter gene imaging. Cellular and humoral responses to reporter gene derived proteins can result in clearance of transduced cells, abrogating the possibility for repeated non-invasive imaging 57. The major strategy to circumvent this issue is to induce immunologic tolerance to these new antigens, usually by immunosuppression, the use of different routes of administration or optimization of the gene promoter 57, 58. Nevertheless, in the context of cancer gene therapy or GDEPT applications perhaps a pro-inflammatory response in the tumor microenvironment could be of short term benefit, providing an influx of immune cells and the possibility of an anti-tumor immune response 59.

Although NfsA_Ec demonstrates activity with all of the agents (listed in Table 3), particularly EF5, ^18^F-EF5 faces significant commercial development challenges due to the requirement for ^18^F_2_ gas for radiolabeling by electrophilic fluorination 60. Most clinical imaging sites routinely utilize the nucleophilic substitution method for routine radiolabeling of ^18^F-FDG 61. Thus, it is likely that successful development of NfsA_Ec based PET imaging will involve probes that can be radiolabeled in the same manner as ^18^F-FDG, for example ^18^F-HX4 62. We therefore selected ^18^F-HX4 to exemplify the preclinical proof-of-principle, particularly as it has other desirable properties (e.g. faster clearance from normal tissues) relative to other radiolabeled 2-NI PET probes such as F-miso 27, 31, 63. Successful ^18^F-HX4 microPET imaging of NfsA_Ec expressing tumors indicated that NfsA_Ec-mediated metabolism of 2-NI probes has the potential for non-invasive monitoring of NfsA_Ec by using the clinically relevant PET imaging tool. However, background hypoxic signal will always be a confounding issue with the use of 2-NIs in this context. Mitigation for this could include the selection of non-hypoxic individual tumors, carbogen breathing to suppress hypoxic signal, and pre/post imaging correction strategies. We are currently developing novel NTR-selective 2-NI probe analogues to overcome this limitation by virtue of being refractory to hypoxic metabolism 64.

## Materials and Methods

### Preparation and storage of chemicals

For *in vitro* studies, PR-104A was synthesized, purified and stored as previously reported 65, 66. Pimonidazole (Hypoxyprobe-1, Chemicon International), HX4 (synthesized at the ACSRC using published methods 67), F-Misonidazole (ABX Company, Germany), RSU-1069 and RB6145 (synthesized at the ACSRC using published methods 68), EF5 and EF3 (generous gifts from Professor Cameron Koch, University of Pennsylvania) were either dissolved in DMSO and stored at -80 °C or dissolved in α-MEM immediately prior to commencing the experiment. For *in vivo* studies, PR-104 (PR-104 sodium salt lyophilized with mannitol) was supplied by Proacta Inc., and reconstituted in 2 mL water before dilution in PBS. EF5 was dissolved in phosphate buffered saline, and pimonidazole was dissolved in saline. The ^18^F-HX4 PET tracer was delivered from the VU University Medical Center Amsterdam, the Netherlands and synthesized as previously described 43. Radiochemical purity was higher than 95% and specific activity was 32.8 ± 0.79 GBq/µmol.

### *E. coli* SOS, NADPH depletion, and IC_50_ assays

SOS assays were performed as previously described using the strain SOS-R2 35. Briefly, test enzyme over-expression was induced and exponential growth phase bacteria were exposed to either 0 µM, 2 µM or 4 µM RB6145 for four hours prior to quantification of the SOS response induction by β-galactosidase assay. Data were calculated as fold induction of Miller units recorded for the drug exposed cultures relative to the unchallenged replicates. NADPH consumption assays were performed by nitroblue tetrazolium/phenazine methosulphate (NBT/PMS) assay as previously described 34. *E. coli* IC_50_ assays were performed in SOS-R2 host cells as previously described 35, with the substitution of CB1954 from that study with a two-fold dilution series of each 2-NI test compound. IC_50_ values were calculated as the 2-NI concentration required to reduce the turbidity of a nitroreductase expressing culture to 50% that of an unchallenged control, using GraphPad Prism 6 (GraphPad Software Inc. La Jolla, CA, USA).

### Protein purification and steady state kinetics

Recombinant His_6_-tagged NTRs were purified post-expression from plasmid pET28a(+) by nickel-affinity chromatography (Novagen, Merck, Darmstadt, Germany). FMN cofactors (or FAD in the case of MdaB) were reconstituted and proteins were desalted, quantified, assessed for purity and stored as previously described 34. The molar extinction coefficient was determined for each 2-NI substrate at 340 nm, accounting for the oxidation of two moles of NDAPH oxidized per mole of 2-NI substrate converted to hydroxylamine. For this, 100 µM of 2-NI was incubated with 300 µM NADPH and excess purified NfsA_Ec for 30 minutes, with the low-level intrinsic NADPH oxidase activity of NfsA_Ec ensuring the complete oxidation of all NADPH remaining in the cuvette within the timeframe of the experiment. On this basis the 340 nm extinction coefficients (M^-1^ cm^-1^) for each 2-NI test compound were calculated as EF5, 19 000; EF3, 19 100; F-Miso, 18 900; pimonidazole, 18 600; RB6145, 19 500; HX4, 19 000. Steady-state enzyme kinetics for purified nitroreductase candidates were assessed as previously described 34 over a 2-NI concentration range of 6.25-1200 µM.

### Cell lines and candidate gene expression

HCT116 WT and H1299 WT cells were purchased from the ATCC (Manassas, VA, USA). HCT116 cell lines over-expressing POR and the *E. coli* nitroreductases had been previously generated and validated for candidate gene expression 34, 35, 69, in addition to the H1299 cell line over-expressing NfsA_Ec 54 (Figure S1). Cell cultures were re-established from STR-authenticated frozen stocks every 3 months and were confirmed to be mycoplasma free by PCR enzyme-linked immunosorbent assay (ELISA) (Roche Diagnostics Corp, Basel, Switzerland). Cell lines were cultured in α-MEM using a humidified incubator (37 °C, 5% CO_2_) as previously described 70, 71 for a maximum of 12 weeks. Harvested cells were counted using an electronic particle counter (Z2 Coulter Particle Analyzer, Beckman Coulter, Florida, USA).

### Anti-proliferative (IC_50_) tumor cell assay

The anti-proliferative IC_50_ was determined as the concentration of prodrug required for 50% inhibition of cell growth, following an 18 hour drug exposure and five days regrowth in the absence of drug. Assays were performed under oxic conditions as described previously 70, 72.

### ^14^C covalent binding assay

The ^14^C-EF5 binding assay was performed as described previously 51. In brief, 1x10^5^ cells/well of a 96-well plate were incubated with 2 mM ^14^C-EF5 under oxic or anoxic conditions for 4 h. Reduced and bound ^14^C-EF5 adducts were measured in the trichloroacetic acid fixed cell pellets using a scintillation counter.

### 3D multicellular layer assay

Multicellular layers (MCLs) were grown and assays performed as previously reported 71. MCLs were exposed to drug for five hours and flushed continuously during drug exposure with 5% CO_2_/95% O_2_ gas to minimize hypoxia and therefore drug activation by endogenous one electron reductases 52. MCL inserts were then dissociated with trypsin, counted, and resuspended in fresh media for use in further experiments.

### Flow cytometry

Following treatment (either *in vitro* culture or *ex vivo* cells following enzymatic dissociation), 1x10^6^ cells were fixed with 4% (w/v) paraformaldehyde (pH 7.4) in PBS for one hour at room temperature. Cells were permeabilized in 0.2% (v/v) Triton X-100 in PBS for 30 minutes at room temperature, before blocking in 10% BSA (w/v) in PBS for 30 minutes at room temperature. Cells were then suspended in primary antibody diluted in 1% BSA (w/v) in PBS for 2 hours at 37 °C (anti-EF5 mouse monoclonal conjugated with Alexa-488 or Cy5 and diluted to 100 μg/mL, a gift from Professor Cameron Koch, University of Pennsylvania, or Hypoxyprobe-1 mouse monoclonal (anti-pimonidazole, Chemicon International) conjugated with FITC and diluted 1:100). Cells were washed 3 times in 1% BSA (w/v) in PBS to remove antibody and resuspended in a final volume of 500 μL. Single cells were then analyzed on a Becton Dickinson FACscan flow cytometer.

### Immunohistochemistry

*In vitro:* 1x10^6^ cells were fixed with 4% (w/v) paraformaldehyde (pH 7.4) in PBS for one hour at room temperature. Cells were permeabilized in 0.2% (v/v) Triton X-100 in PBS for 30 minutes at room temperature, before blocking in 10% BSA (w/v) in PBS for 30 minutes at room temperature. Cells were then suspended in primary antibody diluted in 1% BSA (w/v) in PBS for 2 hours at 37 °C (anti-EF5 mouse monoclonal conjugated with Alexa-488 or Cy5 and diluted to 100 μg/mL, a gift from Professor Cameron Koch, University of Pennsylvania, or Hypoxyprobe-1 mouse monoclonal (anti-pimonidazole, Chemicon International) conjugated with FITC and diluted 1:100). Cells were washed 3 times in 1% BSA (w/v) in PBS to remove antibody and four drops of cell suspension was then added to a cytospin chamber containing a poly-L-lysine coated slide and centrifuged for 5 minutes at low acceleration in a cytospinner (Shandon Cytospin 2). Cells were left to dry for 30 minutes then mounted with a coverslip using Prolong Gold anti-fade reagent (Invitrogen, USA) and sealed with nail polish.

*In vivo:* NIH-III mice were treated with 120 mg/kg EF5 24 hours prior to tumor excision. Tumors were excised, cut in half and fixed in 10% (v/v) formalin in PBS for 48 hours, before being transferred to 70% (v/v) ethanol and embedded in paraffin. Sections (5 μM) were cut and mounted onto poly-L-lysine coated slides, before being heat fixed for one hour at 60 °C. Sections were then de-waxed, rehydrated, washed in MilliQ water, and rinsed in 0.01 M Tris buffered saline (TBS, pH 7.4). Antigen retrieval was achieved by boiling samples in 10 mM sodium citrate buffer (pH 6) for 25 minutes. Sections were washed with TBS containing 0.1% (v/v) Tween-20 (TBS-T) and blocked with 1% mouse serum in TBS. After rinsing, samples were incubated for two hours at room temperature with 100 μg/mL anti-EF5 primary antibody (Alexa-488 conjugated) diluted in PBS containing 0.2% (v/v) Tween-20. After rinsing in TBS-T coverslips were mounted with Prolong Gold anti-fade reagent (Invitrogen, USA) and sealed with nail polish. Following completion of fluorescent imaging, coverslips were removed and the sections were stained with hematoxylin and eosin. Slides were viewed on an inverted fluorescent microscope (Eclipse TE-2000E, Nikon, Japan) and images were analyzed using Adobe Photoshop software (Adobe Photoshop 4.0).

### Animal husbandry

All tumor excision assay and growth delay experiments were performed in accordance with local institutional guidelines for animal welfare and were approved by the Animal Ethical Committee of the University of Auckland. Specific pathogen-free homozygous NIH-III (NIH-Lyst^bg^ Foxn1^nu^ Btk^xid^) nude mice were obtained from Charles River Laboratories (Wilmington, MA, USA) and bred in Vernon Jansen Unit (University of Auckland). They were supplied at 7-9 weeks of age. Mice were housed in groups of ≤ 6 in Techniplast microisolator cages with a 12 hour light/dark cycle, and were fed a standard rodent diet (Harlan Teklad diet 2018i) and water *ad libitum*. All animals were uniquely identifiable by ear tag number and weighed 18 to 25 g at the time of the experiment.

### Tumor excision assay

Tumors were inoculated onto the lateral flank of NIH-III nude mice by subcutaneous injection of 10^7^ cells. Tumor-bearing mice were randomized to treatment groups when tumors reached treatment size (10-12 mm diameter) and administered a single dose of drug (PR-104 = 562 µmol/kg, EF5 = 30 mg/kg) by intraperitoneal injection. After the appropriate exposure time, tumors were excised and homogenized using sterile scissors and an enzyme mix (2.5 mg/mL pronase, 1 mg/mL collagenase and 0.2 mg/mL DNAase in αMEM containing 10% (v/v) fetal bovine serum and 1% (v/v) penicillin/streptomycin). Tumor samples were incubated at 37 °C on a magnetic stirrer, before part of the suspension was collected and resuspended in fresh α-MEM for counting. Serial dilutions of 10^5^-10^2^ cells were plated on 60 mm dishes in triplicate, and grown for 10 days before staining with methylene blue (2 g/L in 50% aqueous alcohol). Colonies containing > 50 cells were counted as clonogenic survivors. Plating efficiency and log cell kill relative to untreated controls was then calculated.

### Tumor growth delay

Tumor-bearing mice were randomized to treatment groups when tumors reached treatment size (300-350 mm^3^) and injected with a single intraperitoneal dose of PR-104 (1000 µmol/kg) or vehicle. Tumor size and body weights were measured 2-3 times per week. Tumor volume was calculated as *L* x *w^2^*x π/6 where *L* is the major axis and* w* is the perpendicular minor axis. Animals were culled when the tumor volume had increased four-fold relative to pre-treatment volume (RTV4, survival endpoint) or if body weight loss exceeded 20% of the pre-treatment value. Kaplan Meier plots were constructed to calculate median time to endpoint. Treatment efficacy was assessed by comparing the median survival time with untreated animals using the Log-rank test P-test (Sigmaplot version 14.0).

### PET imaging and analysis

All PET experiments were in accordance with local institutional guidelines for animal welfare and were approved by the Animal Ethical Committee of Maastricht University (2012-102). Exponentially growing human colorectal (HCT116) or lung (H1299) carcinoma cells (5 × 10^6^) with or without NfsA_Ec overexpression were resuspended in 50 µl Basement Membrane Matrix (Matrigel^TM^ BD Biosciences) and inoculated subcutaneously into the lateral flank of NIH-III nude mice (age 8 weeks). Tumors were measured using a Vernier Caliper in three orthogonal tumor diameters (A, B and C), each corrected for the thickness of the skin, and tumor volumes were calculated based on the formula A × B × C × π/6. Animals were injected with ^18^F-HX4 (3.15 ± 1.30 MBq) via the lateral tail vein at a tumor volume of 525 ± 296 mm³. This is a diagnostic amount of probe (100 µCi), as previously described 73-75. Isoflurane inhalation anesthesia (2.5% isoflurane in pressured air at a flow rate of 3 L/min) was used during all animal experiments.

PET imaging was performed on a Focus 120 MicroPET (Concorde Microsystems Inc., Knoxville) with an axial field of view of 7.6 cm and a resolution of approximately 1.4 mm. A 10-min emission scan was performed on 2 hours post tracer injection with correction for random counts, dead time and decay. Data were acquired using three-dimensional list-mode settings using an energy window of 350-750 keV and a coincidence window of 6 ns. After sinogramming, data were reconstructed iteratively using 3D-OSEM (Fourier rebinning, 16 subsets, 10 iterations and 5 EM iterations, Gaussian smooth 1.73 mm). Images were visualized with ASIPro VM software (version 6.3.3.0; Concorde Microsystems Inc., Knoxville, TN). For each data set, three-dimensional regions of interest (3D-ROIs) were manually drawn over the heart outflow area (representing blood pool) and tumors. Maximum activity data (in Bq/ml) of the voxels, corrected for ^18^F decay towards injection, within the ROIs were obtained and tumor-to-blood uptake ratios were calculated.

### Statistical analysis

Statistical analyses were performed using Sigmaplot version 11.0 (Systat Software) or GraphPad Prism versions 5.02 and 5.03 for Windows (GraphPad Software, 2009). For all tests, *P* < 0.05 was considered significant.

## Supplementary Material

Supplementary figures and tables.

## Figures and Tables

**Table 1:** Micromolar IC_50_ values for NTR-expressing *E. coli* strains exposed to a panel of 2-nitroimidazole compounds

	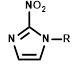					
R =	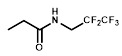	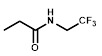	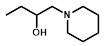	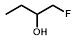	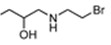	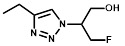
NTR	EF5	EF3	Pimonidazole	F-Miso	RB6145	HX4
NfsA	140 ± 9	83 ± 4	180 ± 5	110 ± 0.5	14 ± 1	280 ± 20
NfsB	>1000	690 ± 80	>1000	860 ± 20	>250	670 ± 30
AzoR	>1000	>1000	>1000	>1000	>250	>1500
NemA	>1000	>1000	>1000	>1000	>250	>1500
Yief	>1000	>1000	>1000	>1000	>250	>1500
MdaB	>1000	>1000	>1000	>1000	>250	>1500
WrbA	>1000	>1000	>1000	>1000	>250	>1500
YdjA	>1000	>1000	>1000	>1000	>250	>1500
YcdI	>1000	>1000	>1000	>1000	>250	>1500
YcaK	>1000	>1000	>1000	>1000	>250	>1500
KefF	>1000	>1000	>1000	>1000	>250	>1500
Empty	>1000	>1000	>1000	>1000	>250	>1500

**Table 2:** Steady state kinetic parameters for reduction 2-nitroimidazole substrates by purified His_6_-tagged nitroreductase candidates

Nitroreductase^a^	Compound	*k*_cat_^b^ (s-1)	*K_M_*^b^ (µM)	*k*_cat_/*K_M_ (mM^-1^ s^-1^)*
NfsA	EF5	8.9 ± 0.3	160 ± 20	56 ± 8
	EF3	10.7 ± 0.4	160 ± 21	66 ± 9
	F-misonidazole (F-miso)	8.8 ± 0.6	480 ± 70	18 ± 3
	HX-4	13.5 ± 0.6	180 ± 25	75 ± 11
	Pimonidazole (Pimo)	11.3 ± 0.5	240 ± 26	47 ± 5
	RB6145	2.7 ± 0.2	200 ± 33	14 ± 2
NfsB	EF5	0.4 ± 0.1	1800 ± 840	0.2 ± 0.1
	EF3	1.1 ± 0.5	5800 ± 3700	0.2 ± 0.1
	F-misonidazole	2.1 ± 0.1	5200 ± 3100	0.4 ± 0.3
	HX-4	29.5 ± 0.1	1100 ± 160	26 ± 4
	Pimonidazole, RB6145	No detectable activity		
AzoR	EF5, EF3, F-miso, HX4, Pimo, RB6145	No detectable activity		
NemA	EF5, EF3, F-miso, HX4, Pimo, RB6145	No detectable activity		
Yief	EF5, EF3, F-miso, HX4, Pimo, RB6145	No detectable activity		
MdaB	EF5, EF3, F-miso, HX4, Pimo, RB6145	No detectable activity		
WrbA	EF5, EF3, F-miso, HX4, Pimo, RB6145	No detectable activity		

a: YcdI and KefF were recovered in the insoluble fraction and were not able to be purified; b: Apparent KM and kcat as determined at 200 mM NADPH.

**Table 3:** Comparative sensitivity of nitroreductase-expressing HCT116 cells to HCT116 WT cells for various nitro(hetero)aromatic compounds

	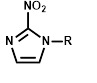						
	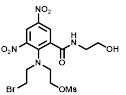	**R =**					
		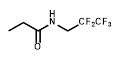	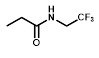	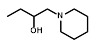	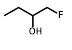	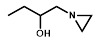	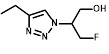
Cell Line	PR-104A	EF5	EF3	Pimo	F-miso	RSU1069	HX4
NfsA_Ec	985	1600	1050	71	690	108	52
NfsB_Ec	1643	57	60	2	203	13	103
YcaK_Ec	1	1	1	1	1	1	1
YieF_Ec	2	1	2	1	1	2	1
AzoR_Ec	3	1	1	1	1	2	3
MdaB_Ec	33	1	1	1	1	2	1
WrbA_Ec	4	2	1	1	1	2	1
KefF_Ec	3	1	1	1	1	1	1
YcdI_Ec	1	1	1	1	1	1	1
YdjA_Ec	1	2	2	1	1	1	1
NemA_Ec	14	4	8	2	28	5	4

Cell lines were exposed to compounds for 18 hours at a range of concentrations followed by 5 days growth in drug-free media. IC_50_ values were determined as the concentration required to inhibit cell growth by 50% of untreated controls. Values in the table represent the fold change in IC_50_ of the stated cell line relative to HCT116 WT cells. Raw IC_50_ values are provided in Supplementary table 1.

## Abbreviations

FDA: U.S. food and drug administration
GDEPT: gene-directed enzyme-prodrug therapy
GCV: ganciclovir
hNIS: human sodium iodide symporter
HSV1-tk: herpes simplex virus 1 thymidine kinase
MCL: multicellular layer
2-NI: 2-nitroimidazole
NTR: nitroreductase
PET: positron emission tomography
3D: three dimensions
TGD: tumor growth delay
WT: wild type
