# Supplementary material for: E. coli nitroreductase NfsA is a reporter gene for non-invasive PET imaging in cancer gene therapy applications
Source: Theranostics. 2020 Aug 21;10(23):10548–62. doi: 10.7150/thno.46826 (PMC7482819; doi:10.7150/thno.46826)

Supplementary file for article:

***E. coli* nitroreductase NfsA is a reporter gene for non-invasive PET imaging in cancer gene therapy applications.**

Alexandra Marie Mowday et al. Theranostics, 2020, doi:10.7150/thno.46826.

Table S1. Cell lines stably transfected with reductases were exposed to compounds for 18 hours at a range of concentrations followed by 5 days growth in drug-free media. Cell line IC_{50} values (μM) were determined as the concentration required to inhibit cell growth by 50% of untreated controls. Standard errors are omitted for clarity.

Cell line	PR-104A	EF5	EF3	Pimonidazole	F-Misonidazole	RSU-1069	HX4
**WT**	23	8000	8400	1200	6900	43	2060
**POR**	31	4000	3400	900	4100	54	2840
**NfsA**	0.024	5	8	17	10	0.4	40
**NfsB**	0.014	140	140	800	34	3	20
**YcaK**	23	6300	7400	1200	6000	45	2420
**YieF**	12	5400	5500	900	7100	23	2700
**AzoR**	7	6400	7600	1300	4900	28	800
**MdaB**	0.7	7400	7200	1200	6100	22	5400
**WrbA**	6	4000	7000	1900	8000	25	2540
**KefF**	8	6300	6300	1300	7000	44	4000
**YcdI**	23	6300	7400	1300	6200	52	3440
**YdjA**	25	5000	4400	1400	4800	51	1840
**NemA**	1.7	2070	1100	773	248	8.2	500

Figure S1. A) Plasmid map of the F279-V5 expression vector containing NfsA_Ec and B) Western blot analysis of protein expression in HCT116 and H1299 stable cell lines. Stable HCT116 or H1299 cell lines expressing NfsA_Ec were created using the FuGENE 6 transfection reagent and subsequent puromycin selection. Surviving cells were combined to form pooled cell populations and successful integration of the expression plasmid into cells was confirmed by western blot analysis using a polyclonal anti-NfsA_Ec antibody.

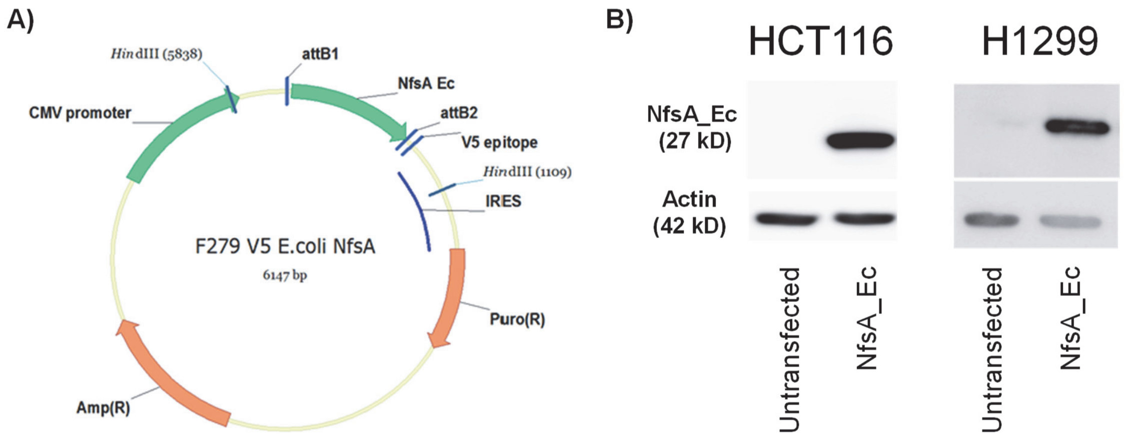

Figure S2. Procedure to 'phenotype' NfsA_Ec-expressing cells following exposure to EF5 in mixed cell culture conditions. A) Summary of the 'NfsA_Ec phenotyping procedure'. B) and C) Example of the gating mechanism used to determine the percentage and fluorescence of NfsA_Ec-expressing cells. Cells were treated with EF5 in 3D conditions (MCL or tumour xenograft). Following trypsinisation, 1x10^6 cells were plated as a single layer and exposed to 20 µM pimonidazole for one hour at 37 °C in air. EF5 adducts were detected using a specific antibody conjugated to CY5 and pimonidazole adducts were detected using a specific antibody conjugated to FITC.

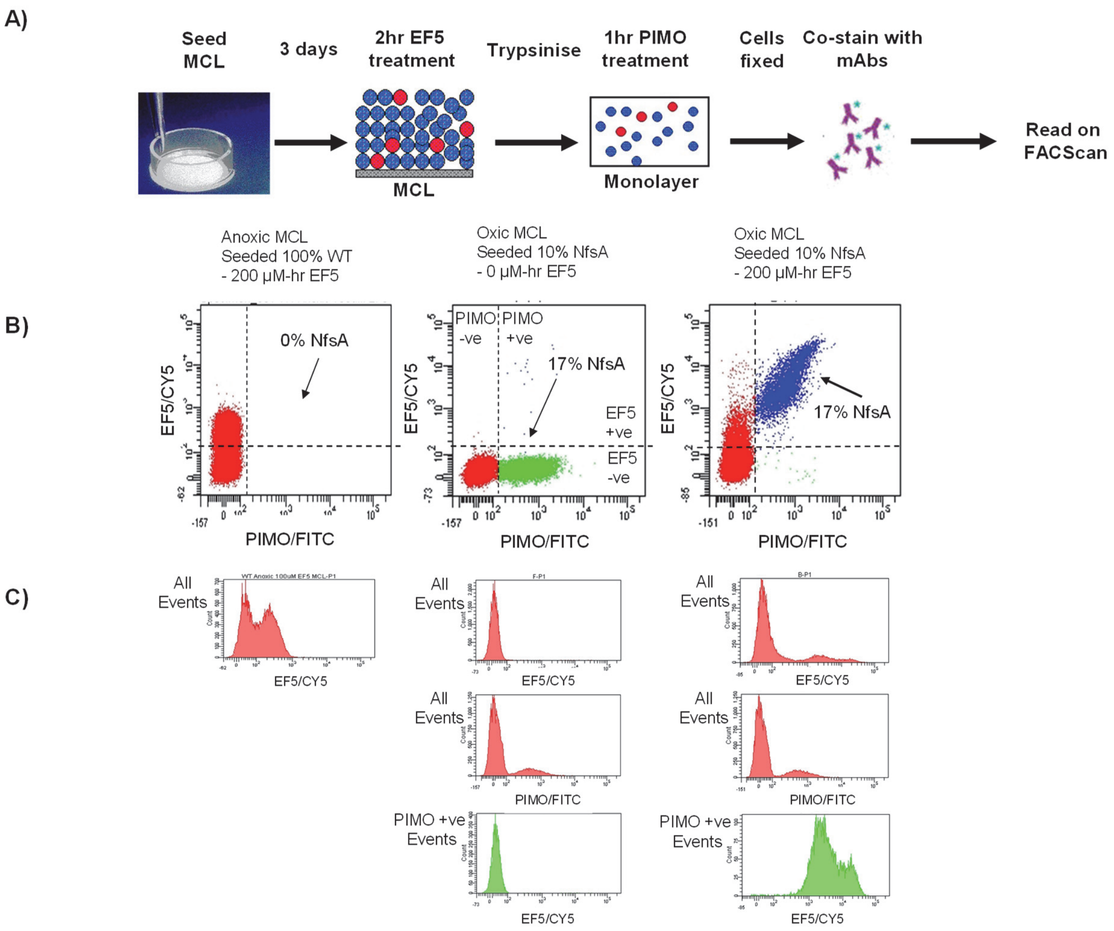

Figure S3. Absolute tumor uptake values in the HCT116 and H1299 tumor models two hours after injection with ^{18}F-HX4.

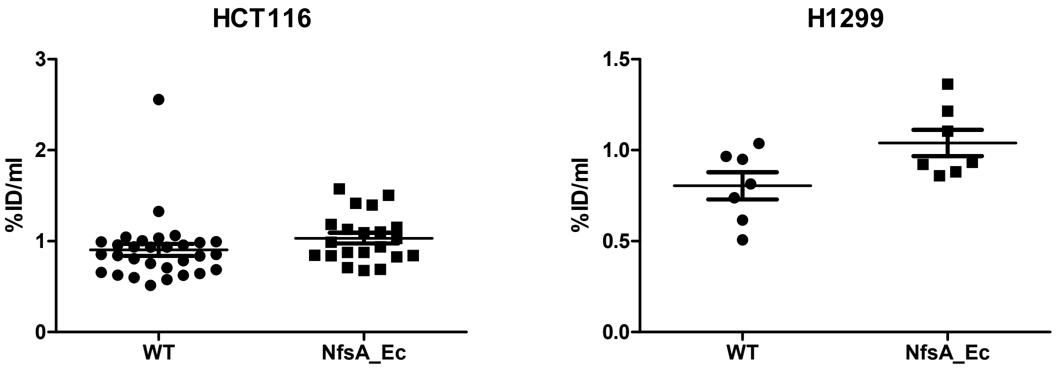

Supplement: Supplementary file 1 — Supplementary figures and tables. [file thnov10p10548s1.pdf]
